# Are sleep disturbances modifiable risk factors for mild cognitive impairment and dementia? A systematic review of large studies

**DOI:** 10.1007/s11325-025-03421-0

**Published:** 2025-08-09

**Authors:** Giusy Bergamo, Claudio Liguori

**Affiliations:** 1 Department of Systems Medicine, University of Rome Tor Vergata, Rome, Italy; 2 Neurology Unit, University Hospital of Rome Tor Vergata, Rome, Italy

**Keywords:** Sleep quality, Sleep duration, Alzheimer’s disease, Actigraphy, Sleep, Cognitive dysfunction, Sleep wake disorders, Alzheimer's disease

## Abstract

Studies have shown a connection between sleep disorders, mild cognitive impairment and dementia. In this context, the present systematic review aimed to determine in large studies whether sleep disturbances are modifiable risk factors for cognitive decline. Following the application of inclusion and exclusion criteria, this systematic review selected 15 studies, with large cohort of subjects included (more than 1000 participants), who were longitudinally observed. Studies predominantly used questionnaires and interviews to collect subjective data on sleep. Eleven studies were based on subjective measurements, one was based on the International Classification of Diseases − 9th Edition diagnosis codes, and three based on objective actigraphic measurements. No study used polysomnographic assessments for the evaluation of sleep disorders.

The results of this systematic review showed that extreme sleep durations (either too short or too long), daytime sleepiness, circadian sleep-wake cycle disruption, and variation in sleep patterns are factors associated with an increased risk of developing cognitive decline and dementia. Actigraphy, as an objective instrument for monitoring the sleep-wake rhythm, provided further insights into the association between sleep problems and longitudinal cognitive decline. These findings emphasize the strong connection between sleep disturbances, circadian rhythm, and the risk of developing dementia and Alzheimer’s disease (AD). Sleep disorders may serve as an early indicator for cognitive decline, also considering that they may represent a modifiable risk factor for dementia. Therefore, recognition and treatment of sleep problems should be included in the prevention strategies against cognitive decline, opening up new opportunities for the prevention and treatment of cognitive impairment and AD.

## Introduction

Epidemiological studies conducted in Italy showed that mild cognitive impairment (MCI) and dementia have prevalence rates of 16.1% and 9.5%, respectively, with dementia incidence at 7.63 per 1,000 person per year [1]. On a global scale, the World Alzheimer Report 2015 estimated 46.8 million individuals were affected by dementia in 2015, with projections reaching 131.5 million by 2050 [2]. However, a comprehensive review noted considerable differences in prevalence and incidence estimates for MCI and related conditions across studies, underscoring the need for consistent definitions to better understand the disease burden [3].

Recent Alzheimer’s Disease (AD) clinical trials focused on pathophysiology-based, disease-modifying therapies and the recruitment of participants at earlier stages of the disease [4]. This change stems from mixed results in anti-amyloid trials and the development of new drugs targeting amyloid and/or tau pathology, neuroinflammation, and neurotransmission systems [5]. Recent advancements in AD treatment have led to the Food and Drug Administration (FDA) approval of the anti-amyloid-β monoclonal antibodies aducanumab and lecanemab; moreover, donanemab is showing promising results in the ongoing clinical trial [6]. However, the efficacy of this drug class remains under evaluation, and tau-targeted therapies are still being investigated [5]. Ongoing Phase III trials evaluating safety and efficacy of other anti-amyloid drugs are expected to further fuel the evidence about their role on AD prevention and treatment [7]. While these advancements are promising, questions persist regarding their clinical effectiveness in real-world settings, associated treatment risks, and healthcare resource implications [8]. The field continues to evolve, emphasizing the importance of basic research on AD pathophysiology, prevention, and early intervention [4].

Recent research emphasized the bidirectional relationship between AD and circadian sleep-wake rhythm disturbances. Sleep-wake irregularities may occur in preclinical AD, potentially acting as an early indicator of the disease [9, 10]. Consistently, the circadian sleep-wake dysfunction might contribute to AD onset, although the causal link remains unclear in the early stages of the disease [11]. Common mechanisms connecting these processes include neuroinflammation, neurodegeneration, and circadian desynchronization [12]. Currently, literature findings highlighted that, on the one hand, sleep disturbances can worsen AD progression and, on the other hand, AD pathology can disrupt the sleep-wake rhythm [9]. Identifying sleep-specific symptoms in AD could enable their earlier diagnosis and treatments [12]. Although different treatments have been investigated in improving the sleep-wake cycle in patients with MCI or AD, more extensive research is required to evaluate the potential of mitigating AD progression through treatment of concomitant sleep disorders [12]. Innovative treatments targeting inflammation and metabolic changes in AD may help restoring the circadian sleep-wake rhythm, potentially improving immunological, metabolic, and behavioral functions [12].

Current studies emphasize the importance of sleep quality in preserving cognitive function and preventing dementia. Sleep problems, including its fragmentation, abnormal duration, and conditions such as sleep-disordered breathing (SDB) have been linked to a higher risk of cognitive decline and dementia [13, 14]. Previous researches demonstrated that individuals with MCI and AD can often present poor sleep quality, REM sleep impairment, reduced sleep efficiency, and increased sleep latency [15]. Although sleep problems are common in patients with MCI and dementia, they are often overlooked and inadequately treated [16].

Therefore, the primary objective of the present systematic review was to define the alteration of subjective or objective sleep parameters associated with the increased risk of cognitive decline and dementia at the longitudinal observation. The secondary objective was to highlight for clinicians the specific sleep problems associated with the risk for cognitive deterioration, emphasizing the need for a regular assessment of sleep quality in patients with cognitive impairment. Such assessments should be integrated into the clinical care of older adults and individuals at risk for dementia to facilitate the early detection and management of sleep disturbances, which could potentially slow or prevent cognitive decline [13, 16].

## Methods

### Search strategy

**Fig. 1:**
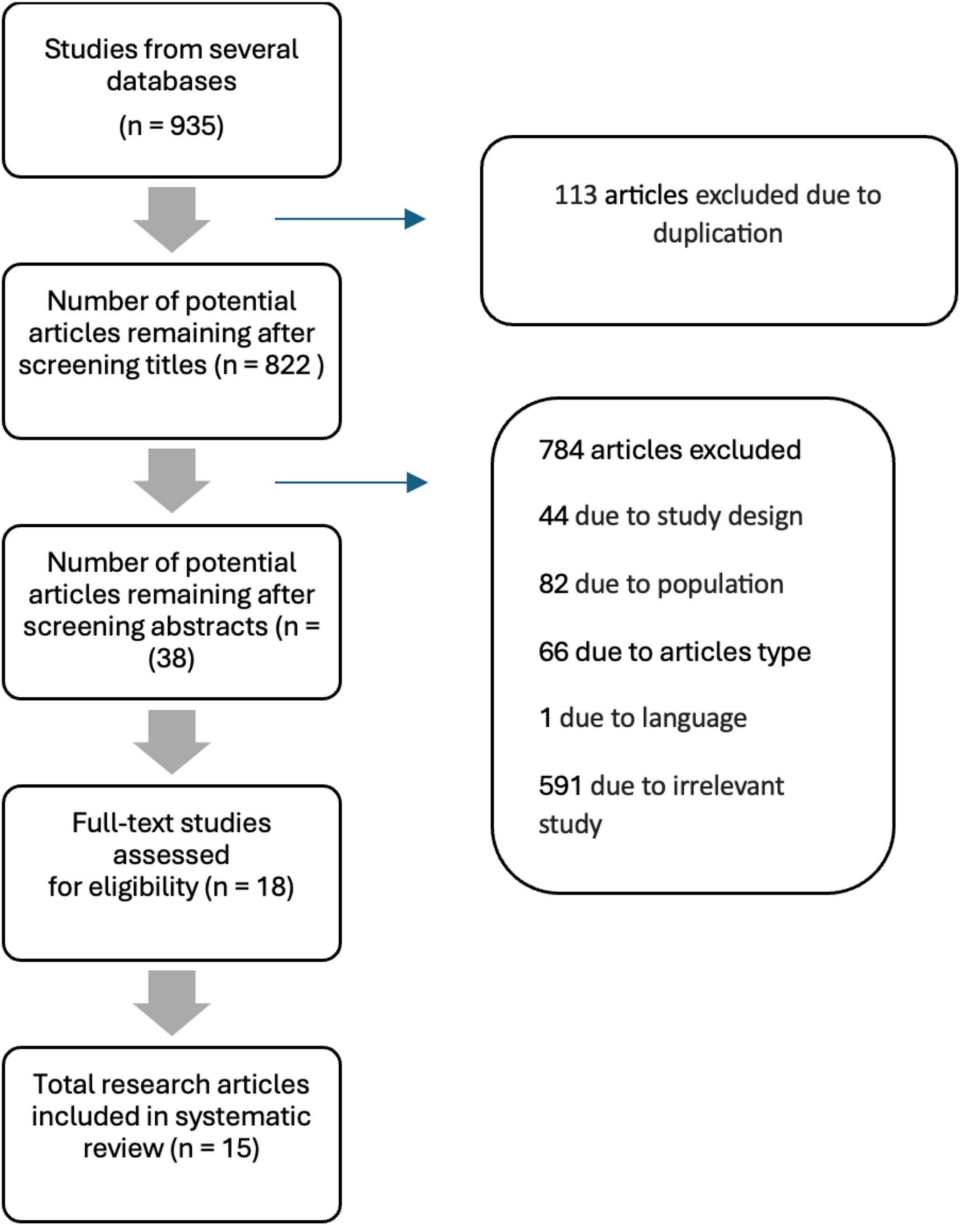
Flow diagram of the study selection process

This systematic review was conducted in accordance with the Preferred Reporting Items for Systematic Reviews and Meta-Analyses (PRISMA, Fig. 1) guidelines. A comprehensive search was carried out in two online research databases, PubMed and Scopus, using the keywords “(insomnia OR sleep duration OR sleep deprivation OR Obstructive Sleep Apnea OR apneas OR circadian rhythm disorders) AND (dementia OR Alzheimer OR Mild cognitive impairment)” for studies published from 2010 onwards. Duplicate records were excluded. Two reviewers (GB and CL) manually screened the remaining records based on the eligibility criteria outlined below. A flowchart of the study selection process is provided in Fig. 1.

### Eligibility criteria

Based on the primary and secondary objectives of the present systematic review, prospective and retrospective studies investigating the incidence of MCI, dementia, and AD in individuals with sleep problems (insomnia, reduced sleep duration or sleep deficiency, Obstructive Sleep Apnea [OSA] or SDB, and circadian sleep-wake rhythm disorders) were selected. Sleep problems had to be evaluated through sleep diaries, medical history, and questionnaires, or objectively assessed through polysomnography and/or actigraphy. Studies included in this analysis required to have a follow-up period of at least two years. Considering the importance of sleep evaluation in the management of patients with cognitive decline, and aiming to ensure a more robust evaluation of the risk of cognitive decline in large populations, only studies with a sample size greater than 1000 participants were included. Additionally, only studies published in English were included in this systematic review (Fig. 1).

### Data extraction

Two reviewers evaluated studies published between January 2010 and December 2023 to identify potential studies for inclusion in this systematic review. The time period was selected based on the updates in diagnostic criteria for MCI, dementia, AD diagnosis, and sleep analysis [17]. The extracted data included: country; sample characteristics (size, average age, proportion of women, and cohort name if available); sleep assessment methods; sleep parameters; methods of cognitive function assessment and/or diagnosis of MCI, dementia, and AD; outcomes; key conclusions.

## Results

A total of 15 studies were included in the final analysis. The flowchart (Fig. 1) shows the number of studies identified, screened, assessed for eligibility, excluded (and reasons for exclusion) and included. This review included only studies with more than 1000 participants, according to the inclusion criteria, and thus most of them relied on subjective data collected via scales, questionnaires, or clinic interviews. Accordingly, of the 15 selected studies, 11 used subjective measurements (questionnaires, sleep diaries, and interviews), 1 used ICD-9 diagnosis codes. Considering the objective measurement that can be used for monitoring sleep and the sleep-wake cycle, 2 studies used actigraphy and 1 study combined actigraphy, sleep diaries, and two items of the Pittsburgh Sleep Quality Index - PSQI - “Time in bed” and “Sleep efficiency”. A brief description of study population, sleep measures and key results of the included studies are summarized in Table 1.

**Table 1:** Main findings of the selected studies

Study	Population	Sleep Measure	Key Results
Beydoun et al. (2021)	Older adults	Self-reported sleep duration	Sleep < 6 h or > 8 h associated with cognitive decline
Chen et al. (2016)	Older women	Self-reported sleep duration	Sleep < 6 h associated with cognitive decline
Chen et al. (2022)	Middle-aged adults	Circadian amplitude by actigraphy	Reduced amplitude associated with increased risk of dementia
Ding et al. (2016)	Older men	Self-reported sleep apnea	Sleep apnea associated with increased risk of dementia
Jackowska and Cadar (2020)	Older adults	Self-reported sleep duration	Sleep < 6 h or > 8 h associated with cognitive decline
Jaussent et al. (2012)	Older adults	Self-reported daytime sleepiness and insomnia	Excessive daytime sleepiness predictive of cognitive decline
Lee et al. (2022)	Older adults	Informant-reported sleep disturbances	Sleep disturbances associated with increased dementia risk
Li et al. (2023)	Older adults	Nap frequency and duration by actigraphy	Longer and more frequent naps associated with increased risk of Alzheimer’s disease
Lysen et al. (2020)	Middle-aged adults	Sleep parameters by actigraphy	Prolonged sleep latency and reduced sleep efficiency associated with increased dementia risk
Nakakubo et al. (2019)	Older adults	Self-reported sleep duration	Sleep < 6 h associated with cognitive decline
Suh et al. (2018)	Older adults	Self-reported sleep quality	Poor sleep quality associated with cognitive decline
Sung et al. (2017)	Older adults	Self-reported non-apnea sleep disorders	Sleep disorders associated with increased dementia risk
van Oostrom et al. (2018)	Middle-aged adults	Self-reported sleep duration	Sleep > 9 h associated with lower cognitive function
Wu et al. (2021)	Older Chinese adults	Self-reported sleep duration	Sleep < 6 h or > 8 h associated with cognitive decline
Xu et al. (2014)	Middle-aged and older Chinese adults	Self-reported sleep duration	Sleep < 6 h or > 8 h associated with lower cognitive function

### Questionnaire-based studies

Subjective sleep measurements were used in eleven studies to evaluate sleep quality in cognitively healthy individuals and patients with MCI, with the main characteristics of these studies summarized in Table 2. The Jenkins Sleep Problems Scale (JSS), the Jenkins Sleep Evaluation Questionnaire (JSEQ), the PSQI were the most used tools for investigating sleep quality and disturbances, among the subjective sleep assessments. Specifically, the JSS consists of four items rated on a six-point Likert scale assessing sleep problems over the past month (not present at all = 0; present in 1–3 days = 1, 4–7 days = 2, 8–14 days = 3, 15–21 days = 4, 22–28 days = 5). The PSQI evaluates seven domains related to nocturnal sleep (sleep quality, sleep latency, sleep duration, habitual sleep efficiency, sleep disturbances, use of sleep medications, and daytime dysfunction related to sleep problems), generating a global score that distinguishes good and poor sleepers based on a cut-off of 5. The JSEQ indicates the number of days individuals can experience problems falling asleep, staying asleep, early awakening, and awakening tired in the previous 30 days; the numbers of days were grouped according to predefined categories, and each category was given a score of 0–5, for a possible total score of 0–20.

**Table 2:** Main characteristics of the selected studies using subjective measurements

Reference	Country	Sample (mean age ± SD)	Cognitive Assessment	Sleep Assessment/Parameters	Outcome/Objective
Jaussent et al. (2012)	France	N:4894	MMSE; BVRT; IST	Self-reported insomnia and daytime sleepiness	Association of sleep complaints with cognitive decline
Xu et al. (2014)	China	N:13888 (63.2 ± 6.4 men; 59.9 ± 6.6 women)	DWRT; MMSE	Sleep duration; daytime napping; insomnia	Association between sleep duration and memory decline
Chen et al. (2016)	USA	N:7444 (70.1 ± 3.8)	3MS; CERAD	Self-reported sleep duration	Associations of cognitive decline and MCI/dementia with sleep duration
Ding et al. (2016)	USA, Canada, Puerto Rico	N:7547 (67.5 ± 5.3)	MIS; CERAD-e; TICS-m; AD8	Self-reported sleep apnea	Sleep apnea and risk of dementia
Sung et al. (2017)	China	N:92079 (48.6 ± 16.2)	ICD-9	ICD-9 sleep disorders	Risk of dementia in patients with NSD
Suh et al. (2018)	South Korea	N:2893	CERAD-K; DST; FAB	PSQI sleep parameters	Sleep disturbances inducing cognitive changes
van Oostrom et al. (2018)	Netherlands	N:2970 (55.2 ± 6.9)	Memory, speed, flexibility, global cognition	Self-reported sleep duration and feeling not rested	Association of sleep with cognitive changes
Nakakubo et al. (2019)	Japan	N:2096	NCGG-FAT; MMSE	Self-reported sleep duration and EDS	Sleep and EDS relation with cognitive decline
Jackowska and Cadar (2020)	England	N:4877	Verbal fluency, memory, orientation	JSS (sleep duration and quality)	Sleep and systemic inflammation effects on cognition
Wu et al. (2021)	China	N:16948 (53.0 ± 6.2)	SM-MMSE	Self-reported sleep duration	Sleep change and cognitive impairment
Beydoun et al. (2021)	USA	N:9518 (74.9 ± 0.08)	Memory, speed, mental status	JSEQ (insomnia symptoms)	Insomnia symptoms and cognitive decline
Lee et al. (2022)	USA	N:8460	CDR	NPI-Q (sleep disturbance)	Informant-reported sleep disturbance and dementia risk

In a study conducted in 2018, Suh et al. [18] analyzed a broad cohort of 2,238 participants with normal cognitive function (NC) and 655 with MCI. In NC individuals, long sleep latency (> 30 min), long sleep duration (≥ 7.95 h), and late mid-sleep time (after 3:00 am) at baseline were associated with higher risk of cognitive decline over a 4-year follow-up period. The odds ratio (OR) was 1.40 for prolonged sleep latency, 1.67 for prolonged sleep duration, and 0.61 for late mid-sleep time [18]. These associations remained significant when the sleep patterns persisted throughout the follow-up period. Conversely, among patients with MCI, by evaluating those who reverted to a NC condition, only prolonged sleep latency was associated with a lower chance of reverting to NC (OR = 0.69) [18]. In summary, sleep problems emerged as potential indicators of developing MCI in NC individuals, whereas only prolonged sleep latency resulted as a sleep problems reducing the possibility of reverting to NC in patients with MCI.

The study by Chen et al. (2016) [19] examined the association between sleep duration and risk of MCI and dementia in 7,444 women aged 65 to 80 years old. From the analysis of the study findings, a V-shaped association was observed between sleep duration and longitudinal risk of developing MCI or dementia. In particular, both short (≤ 6 h/night) and long (≥ 8 h/night) sleep durations were associated with higher risk of conversion to dementia compared to intermediate, normal, sleep time (set at 7 h per night). Specifically, short sleepers had a 36% higher risk and long sleepers - without cardiovascular disease - had a 27% higher risk of developing MCI or dementia at the longitudinal observation [19].

Expanding on the link between sleep duration and cognition, Jackowska and Cadar [20] included in their study 4,877 participants to assess the relationship between sleep duration and cognitive performance over time. Sleep duration, a measure of average self-reported number of hours of sleep on a weeknight, was categorized into four groups: “≤6 hours” and “6–7 hours” (short sleep duration), “7–8 hours” (optimal sleep duration), and “>8 hours” (long sleep duration). Among men (mean age of 65.4 ± 8.8 years), both short (≤ 6 h and 6–7 h) and long sleep durations (> 8 h) at baseline were associated with a higher risk of presenting lower scores on verbal memory tests at the 8-year follow-up, particularly in delayed recall test. Conversely, the group of male subjects with optimal sleep duration (7–8 h per night) did not present this risk (total effect: β = −0.263, C.I. −0.506 to − 0.020). No significant associations were observed between sleep measures and cognitive performance at follow-up in women (mean age: 65.8 ± 9.3 years). These findings globally suggested potential sex differences in the relationship between sleep duration and the risk of cognitive decline.

Further supporting the detrimental effects of extreme sleep durations on cognitive performance, Wu et al. (2021) [21] analyzed data from 16,948 Chinese men and women, aged 45 to 74 y.o. at baseline from the Singapore Chinese Health Study cohort. Sleep duration was evaluated at three time points: baseline (1993–1998), follow-up 2 (2006–2010), and follow-up 3 (2014–2016). At follow-up 2, individuals identified as presenting a long sleep duration (≥ 9 h per night) at baseline were associated with a higher risk of cognitive impairment (OR = 1.43; 95% CI: 1.24–1.66), compared to individuals who presented 7 h of sleep per night. At follow-up 3, individuals presenting both long (≥ 9 h per night) and short (≤ 5 h per night) sleep (OR = 2.03 and 1.26, respectively) showed a significant increased risk of presenting cognitive impairment. This finding reinforced the previously documented U-shaped relationship between sleep duration and cognitive performance. Authors also analyzed changes in sleep patterns documented at the different time points of the study and the risks associated with the change from a sleep duration group to another during the longitudinal observation. Specifically, participants who extended their sleep duration from short (≤ 5 h/night) to long (≥ 9 h per night) from baseline to follow-up 2 presented a 2.18-fold higher risk of developing cognitive impairment. Similarly, those who transitioned from a recommended sleep duration (7 h/night) to a long sleep duration had a 1.55-fold increased risk. The greatest risk, notably, was observed among individuals who reduced their sleep from long (≥ 9 h/night) to short (≤ 5 h/night), with 2.93-fold higher odds of developing cognitive impairment.

The study by van Oostrom and colleagues (2018) [22] evaluated the sleep habits of middle-aged adults (mean age 55.2 ± 6.9 years) and documented that a long sleep duration (≥ 9 h) was significantly associated at the follow-up with poorer global cognitive function (β = −0.06; CI: −0.11 to − 0.02), memory abilities (β = −0.09; CI: −0.16 to − 0.01), and cognitive flexibility (β = −0.07; CI: −0.14 to − 0.01). Additionally, both short (≤ 5 h/night) and long (≥ 9 h) sleep durations were associated with lower cognitive processing speed among individuals who frequently reported not feeling rested at awakening. Moreover, long sleepers (≥ 9 h per night) scored lower in cognitive flexibility compared to those with an intermediate/normal sleep duration (7–8 h) [22]. In multivariable linear regression models, changes in self-reported sleep duration were evaluated in association with the cognitive function at follow-up. Although long sleep duration was initially associated with reduced cognitive function at the longitudinal observation, no significant associations were found between changes of sleep duration and changes in cognitive function over time [22]. These findings suggest that, in middle-aged adults, the relationship between sleep duration and cognition may be more complex and distinct from the patterns typically observed in older adults.

Xu et al. (2014) [23] involved in their study 13,888 participants (mean age ± SD: 63.2 ± 6.4 years for men and 59.9 ± 6.6 years for women), with an average follow-up of 4.1 years. Authors investigated the association between both short sleep duration (≤ 5 h/night) and long sleep duration (≥ 10 h/night) with the memory performance, as measured by the Delayed 10-Word Recall Test (DWRT), and the cognitive status, as evaluated by the Mini-Mental State Examination (MMSE), measured at follow-up. The study found that only short sleep duration (≤ 5 h/night) was significantly associated at follow-up with an increased risk of developing memory deficits, defined as a DWRT score < 4. The OR for developing a memory deficit was 1.53 (95% CI: 1.21–1.93) for short sleepers compared to those with normal sleep duration (7 h/night). Compared to a frequency of daytime napping of 1–3 times per week, individuals who presented a daily nap showed a higher risk of memory impairment at follow-up– measured by DWRT (adjusted OR = 1.34 [1.06–1.69]).

Beyond sleep duration and in agreement with the latter results documented by Xu et al. [23], Nakakubo et al. (2019) [24], in their 4-year longitudinal study of 3,151 older adults aged 65 or older, explored the impact of sleep duration and excessive daytime sleepiness (EDS) on cognition. EDS was identified by the Authors when the participants answered “almost always” to the question “How often do you experience daytime sleepiness requiring a nap?”. Conversely, answers “almost always”, “sometimes” and “rarely or never” were not considered as EDS. On the one hand, Authors showed that both short (≤ 6.0 h/night) and long (≥ 9.0 h/night) sleep durations were linked to a higher risk of cognitive decline at follow-up compared to intermediate/normal sleep time (6.1–8.9 h/night). Moreover, incidence of cognitive decline at follow-up significantly varied across the sleep duration groups (short sleep: 15.9% [*n* = 35], intermediate sleep: 11.9% [*n* = 191], long sleep: 20.1% [*n* = 54]; *p* = 0.001). On the other hand,participants with EDS (prevalence: 13.1%) had a significant decline of cognitive performance at follow-up compared to participants without EDS (18.9% vs. 12.5%, *p* = 0.004).

Similarly, Jaussent et al. (2012) [25] showed in a large sample of participants aged 65–85 y.o. that EDS increased the risk for cognitive decline (a 4-point reduction in MMSE scores during the 2-, 4-, and 8-year follow-up) at follow-up by 26% (OR = 1.26, 95% CI = 1.02–1.56). Moreover, in the analysis of baseline data of subjects who developed dementia during the follow-up, EDS contributed with an higher risk (39%). This association remained significant in the fully adjusted model, accounting for confounding factors, including the use of prescribed sleep medications. Unexpectedly, difficulty maintaining sleep was negatively associated with cognitive decline at follow-up [25], a finding the Authors suggested may be confounded by cholinesterase inhibitors treatment during the follow-up period. No significant associations were found between sleep disturbances (components of insomnia and daytime sleepiness) and declines in visual memory performance or verbal fluency over the 8-year follow-up period.

P. Sung et al. (2017) [26] conducted a retrospective study involving patients (mean age 48.6 ± 16.2 y.o.) with non-apnea sleep disorders (NSD), defined as insomnia, hypersomnia, circadian sleep-wake rhythm disorders, other non-organic sleep disorders. Authors found significant higher dementia risk in patients with NSDs compared to subjects without sleep disorders (adjusted HR 1.46; 95% CI: 1.38–1.54; *p* < 0.0001). The risk for dementia was highest within the first year following a NSD diagnosis but remained higher even after 5 years of follow-up compared to those without a NSD diagnosis (adjusted HR 1.44; 95% CI: 1.32–1.57; *p* < 0.0001).

SDB was also examined in the “Prevention of Alzheimer’s Disease with Vitamin E and Selenium” (PREADViSE) study by Ding et al. [27], which investigated the association between self-reported sleep apnea and the risk for dementia in the male population included and aged 62 years and older. Participants initially underwent a cognitive screening with the Memory Impairment Screen (MIS), and those who were positive to memory impairment proceeded to second-level screening (CERAD-e or TICS-m). The study found that, among participants without the apolipoprotein E (ApoE) ɛ4 allele, baseline self-reported SDB was associated with a 66% higher risk of developing dementia at follow-up. In contrast, no additional risk was observed in participants carrying the ApoE ɛ4 allele. Further analysis adjusting for confounding factors showed that self-reported SDB remained significantly associated with an increased risk of presenting dementia at follow-up. Although the cumulative incidence was higher in the SDB group (24.4%) compared to the group without SDB (9.3%), the difference was not statistically significant, possibly due to the limited number of dementia cases documented at follow-up (*p* = 0.14 by log-rank test). In the adjusted model, the history of SDB presented a trend toward significance (HR = 1.44; 95% CI: 0.96–2.17; *p* = 0.08). Specifically, men with SDB tended to show a higher likelihood of developing dementia compared to men without SDB, suggesting that sleep apnea may increase dementia risk, particularly in individuals without the ApoE ɛ4 allele.

Focusing on insomnia, Beydoun et al. (2021) [28] analyzed the Health and Retirement Study (HRS) data and found that severe insomnia symptoms at baseline in participants aged ≥ 65 years were associated with an increased risk of physician-diagnosed memory problems (HR = 1.21, 95% CI: 1.02–1.44) at the follow-up, performed over a 10-year period. Specifically, individuals who experienced an increase in the severity of insomnia symptoms over time had a 41–72% higher risk of presenting physician-diagnosed memory problems and a 45–58% higher risk of dementia diagnosis based on HRS criteria.

Finally, Lee et al. (2022) [29] conducted a longitudinal retrospective cohort study using the uniform dataset collected by the National Alzheimer’s Coordinating Center. The results showed that older adults with sleep disturbances had a higher likelihood of developing dementia during an average follow-up of 4.5 years, particularly among those with normal cognition at baseline (HR 1.56, 95% CI: 1.07–2.27). Furthermore, greater severity of sleep disturbances was correlated with an increased dementia risk (HR 1.40, 95% CI: 1.05–1.86).

### Actigraphic studies

The main characteristics of the selected studies with objective sleep measurements are summarized in Table 3. Lysen and colleagues [30] investigated the association between actigraphy-estimated sleep parameters and 24-hour activity rhythms and the risk of dementia in a cohort prospectively evaluated (mean age 66 ± 8 y.o., 53% women). Their findings showed that longer sleep latency (HR per one standard deviation increase: 1.44, 95%, CI: 1.13–1.83) and more time spent in bed (HR: 1.40, 95% CI: 1.04–1.88) documented at the baseline investigation were associated with a higher risk of developing dementia over a 11.2 year of follow-up. In contrast, higher sleep efficiency (HR 0.72, 95% CI 0.55–0.93) and later “lights-off” times (HR 0.56, 95% CI 0.41–0.76) were associated with a lower risk of developing cognitive decline. Moreover, a later timing of onset of the least active 5 consecutive hours (L5), a non-parametric circadian rhythm analysis parameter, was associated with a reduced risk of dementia during the first 2 years of follow-up (HR: 0.23, 95% CI: 0.09–0.61). Notably, total sleep duration itself was not significantly associated with higher dementia risk (HR: 0.97, 95% CI: 0.74–1.29) or AD risk (HR: 0.92, 95% CI: 0.68–1.26). No associations were observed between circadian sleep-wake rhythm and the higher risk of dementia, even after adjusting for potential confounders such as SDB, number of daytime naps, or ApoE ε4 allele.

**Table 3:** Main characteristics of the selected studies using objective sleep measurements

Reference	Country	Sample (mean age ± SD)	Cognitive Assessment	Sleep Assessment/Parameters	Outcome/Objective
Lysen et al. (2020)	Netherlands	N:1322 (66.1 ± 7.6)	MMSE; CAMDEX; informant interview	Actigraphy and sleep diary parameters	Actigraphy-derived sleep measures and dementia risk
Chen et al. (2022)	UK	N:72242 (62.1 ± 7.8)	ICD codes	Actigraphy (relative amplitude)	Circadian rhythms measures and dementia
Li et al. (2023)	USA	N:1401 (81.42 ± 7.47)	Memory, working memory, perceptual speed, visuospatial ability	Actigraphy device parameters	Daytime napping and AD risk

The study by Chen et al. (2022) [31] analyzed 7-day accelerometry data from 72,242 participants in the UK Biobank (54.9% women; mean age 62.1 y.o.). The authors calculated the relative circadian amplitude, which is a measure of circadian sleep-wake rhythm disruption, and found that individuals with reduced relative amplitude exhibited an increased risk of developing dementia after a median follow-up of approximately 6.1 years (HR 1.22 [95% CI 1.14–1.29], *p* < 0.001).

Finally, Peng Li et al. (2023) [32] documented that older adults tended to take longer and more frequent daytime naps. Using actigraphy, they found that the progression of AD accelerated the age-related increase in nap duration and frequency. Moreover, longer daytime naps were significantly associated with an increased risk of developing AD, with a HR of 1.20 (95% CI: 1.06–1.35; *p* = 0.004) for each one standard deviation increase in nap duration. Similarly, a higher daytime nap frequency was also linked to a greater AD risk (HR:1.23; 95% CI: 1.08–1.39; *p* = 0.001) for each one standard deviation increase in nap frequency. Interestingly, their study identified a bidirectional relationship between excessive daytime napping and cognitive decline. More excessive napping was correlated with worse cognition one year later, while worse cognition was in turn associated with increasing napping in the following year [32].

## Discussion

The identification of sleep problems as a potential risk factor for the development of MCI, dementia and AD is of critical importance. Early dementia diagnosis and targeted intervention aimed at regulating the sleep-wake circadian rhythm may mitigate the future risk of developing cognitive decline. Consistently, this systematic review sought to elucidate the potential role of sleep disturbances as predictors of cognitive impairment, dementia and AD through longitudinal follow-up studies including a large number of participants.

Based on these premises, the results of the 15 studies selected for this review, according to the inclusion and exclusion criteria, present a complex picture of the association between sleep disturbances and cognitive decline in older adults. Different significant results emerged, although some inconsistencies among studies may rely on the heterogenous study designs. In a significant number of studies (11 of 15), sleep quality was investigated solely through subjective measurements. In these studies, questionnaires such as the PSQI, JSS/JSEQ, NPI-Q, and sleep interviews were utilized. Direct comparability of results obtained by the subjective questionnaires suffers from significant variability. Accordingly, operational definitions of “sleep disturbance”, “prolonged sleep latency”, or “daytime sleepiness” can vary significantly in sensitivity and specificity. Actigraphy, as objective instrument able to evaluate the sleep-wake cycle, was adopted only in three studies [30]; [31, 32]. The heterogeneity of the instruments used for identifying sleep disturbances in the different studies does not help in drawing definite conclusions, although it appeared evident that sleep problems are associated with the development of cognitive impairment. In agreement with the results of the present review, sleep problems can be associated with the higher risk of developing cognitive decline, although the neuropathological process at the basis of cognitive decline cannot be defined. In particular, studies based on self-reported measures of total sleep time shows a U-shaped effect. Briefly, both extremely short sleep duration (< 6 h) or excessively prolonged sleep (> 8–9 h) are associated with the increased risk of cognitive decline [19–21, 23, 24, 28]. On the contrary, the actigraphic-defined total sleep time did not show the same significant association with cognitive decline, as showed by Lysen et al. (2020) [30]. Therefore, self-reported measures may over- or under-estimate sleep measures. Considering the study using both the actigraphy and questionnaire-based measures here reviewed, Lysen et al. (2020) [30], documented that a longer latency to fall asleep (> 30 min) was associated with an increased risk of cognitive impairment in both cognitively normal subjects and those with MCI and dementia. The subjective measurement of sleep latency in the study by Suh et al. (2018) [18] showed the same association with the longitudinal development of cognitive decline.

Not only the detrimental effects of sleep problems, but also the beneficial effects of sleep in preventing the cognitive decline have been measured. Better sleep efficiency (HR: 0.72) and later light-off times (HR: 0.56), measured by actigraphic recordings, were associated with a reduced risk of dementia, especially of AD type [30].

The circadian sleep-wake rhythm amplitude, which cannot be easily measured subjectively, was calculated in the actigraphic study by Chen et al. (2022) [31] in more than 72,000 individuals. This study showed that reduced relative amplitude is associated with an increased risk of dementia at follow-up (HR:1.22) [31]. With reference to circadian sleep-wake rhythm, Lysen et al. (2020) [30] suggested that a later L5 phase was protective in the first two years of follow-up against the development of cognitive impairment. These studies suggested that sleep-wake cycle disturbances may be either a risk factor for cognitive impairment or reflect an early manifestation of a neurodegenerative disorder.

Daytime data obtained by questionnaire-based or actigraphic studies were also analyzed. Li et al. (2023) [32] and Chen et al. (2022) [31] showed the association between frequent daytime napping and the development of cognitive decline. In questionnaire-based studies, Xu (2014) et al. [23] documented that EDS is associated with the increased risk of cognitive impairment. As a further result of the study by Li et al. (2023) [32], a bidirectional association between EDS (as measured by the frequency and duration of daytime naps) and cognitive impairment was documented, underlying that cognitive impairment may cause the increase of daytime napping and vice versa.

Sleep patterns undergo significant changes with aging, with modification of both sleep macro and microstructure, particularly from the sixth decade of life onwards. At the macro level, older adults experience lighter and more fragmented sleep, with alterations in sleep architecture [33]. These changes include modifications in the distribution of sleep stages, total sleep time, and sleep efficiency [34]. Moreover, reduction and instability of REM sleep and slow-wave sleep (SWS) seem to characterize sleep architecture of the elderly. At the micro level, age-related changes in sleep are characterized by alterations in brain electrical activity and specific oscillatory events [34]. In particular, arousals impair sleep continuity by producing sleep fragmentation, slow wave activity (SWA) reduces its amplitude, and sleep spindles and K-complexes are less represented and change their morphology. Considering the importance of monitoring sleep in subjects at risk for cognitive impairment, sleep studies can be encouraged for early identifying pathological changes of sleep macro- and micro-structure. However, the design of this review aimed at verifying the longitudinal cognitive decline in large cohort of individuals monitored for their sleep habits cannot permit the inclusion of smaller polysomnographic studies. Polysomnography, the gold standard instrument for sleep architecture analysis, enables the concurrent recording of various physiological parameters (EEG, EMG, EOG, respiratory flow, oxygen saturation), allowing for precise diagnosis of specific sleep disorders like SDB or sleep-realated movement disorders. However, its high cost, invasive nature, and logistical complexity restrict its application in large-scale, longitudinal studies, such as those reviewed here. Instead, three studies employed actigraphy to track sleep-wake patterns for several days in an home environment. While more feasible for studies including large group of subjects and for extended observational periods, actigraphy offers an indirect and less accurate measure of sleep. Questionnaires, used in most of the studies here reviewed, are the most affordable and accessible methods but are even less reliable instruments since they can be more prone to cognitive, emotional, or memory biases—in particular when administered to olders or participants with cognitive impairment.

The evaluation of sleep characteristics and the screening for sleep disorders is critical for preventing the risk of cognitive impairment in the elderly. Accordingly, sleep disturbances have been described in a bidirectional relationship with neurodegenerative processes. On the one hand, sleep problems can interfere with cognitive function and overall brain health, and, on the other hand, the neuropathological changes can produce sleep modifications [35, 36]. However, the role of sleep disorders as modifiable factors for neurodegeneration is currently under evaluation. Only prospective studies that incorporate objective sleep data, serial cognitive evaluations during the follow-up, and biomarkers (e.g., β-amyloid or tau) can determine whether addressing sleep issues might have a genuine preventive or therapeutic effect on AD progression. Clinically, this uncertainty in causality has two key implications. Firstly, it is not yet possible to definitively claim that treating sleep disorders prevents or slows dementia progression. Secondly, early-occurring sleep disorders, such as SDB, insomnia or EDS, could serve as valuable indicators for identifying at-risk individuals before cognitive deficit become apparent. Moreover, coupling the clinical findings with the genetic risk factors for AD, the role of the APOE ε4 allele was evaluated by Ding et al. (2016). The Authors in their study investigated the link between OSA and dementia risk and identified that only individuals without the apoE ε4 allele and presenting OSA were at risk for cognitive decline [27]. This finding implies that in those with less genetic predisposition, sleep disturbances might independently contribute to dementia risk, whereas in apoE ε4 carriers, genetic risk may overshadow the risk associated with sleep disturbances. If confirmed, this interaction would underscore the importance of early identification and treatment of sleep disorders in individuals without a genetic predisposition, as they represent a potentially modifiable risk factor. From this perspective, systematically including sleep assessments in geriatric and neurological practice could enable earlier identification of neurodegenerative risk due to the presence of sleep disorders, guiding further diagnostic evaluations or interventions. Until more definitive etiological evidence is available, integrating the monitoring and management of sleep disorders into elderly care pathways is advisable, not only to enhance quality of life but also as a potential secondary prevention strategy.

This evidence here collected underscores the importance of recognizing sleep problems in older adults, as interventions targeting sleep may contribute to maintaining cognitive performance and well-being, and possibly interfering with the neurodegenerative process in the aging population [36]. Considering the sleep disorder defined according to the International Classification of Sleep Disorder– 3rd Edition, only SBD have been clearly described as a risk factor for dementia, and further studies are needed for identifying insomnia or other sleep disorders as definite risk factors for dementia and AD.

This systematic review presents some limitation to be acknowledged. The selection criteria for this systematic review — specifically, the inclusion of studies with large sample sizes (exceeding 1,000 participants), a follow-up period of at least two years, and both subjective and/or objective sleep assessments — were designed to ensure statistical strength and methodological precision. Nevertheless, these criteria led to the exclusion of smaller studies, controlled clinical trials, or research focusing on specific groups (such as younger individuals, patients with psychiatric conditions, or underrepresented ethnic minorities). As a result, the findings mainly illustrate the link between sleep disorders and cognitive decline in older groups from the general population (≥ 65 years) or those chosen in clinical and observational settings. The effects of sleep on cognitive function can vary with age, which may significantly influence the relationship between sleep disturbance and dementia risk. Moreover, unlike neuropsychological results, which are age- and education-corrected, questionnaires assessing sleep disturbances do not account for age, rendering them nonspecific in the age groups most at risk of neurodegenerative disease onset, such as the elderly. Another limitation can be related to the lack of sex-based differences in the results analyzed. Gender and biological sex, indeed, emerged in the recent literature as a possible potentially modulatory factor in the relationship between sleep parameters and risk of cognitive decline. Jackowska and Cadar (2020) [20] found that the association between sleep duration and memory decline in men, indicating that sex and gender might play a moderating role in cognitive vulnerability related to sleep. However, none of the studies systematically conducted sex-stratified analyses or examined different pathophysiological mechanisms, such as circadian regulation or neuroinflammatory responses to disrupted sleep, that could differ between genders. Finally, the lack of polysomnographic data limited the possibility to analyze sleep’s microstructural components (e.g., slow-wave sleep, SWA, REM sleep), which have been linked to specific cognitive functions and AD. To gain a deeper understanding of how sleep disturbances contribute to cognitive decline, future research should consider multimodal approaches that combine subjective, actigraphic, and polysomnographic measures, albeit possibly in selected subsamples. The limitations of this systematic review mean that the conclusions cannot be directly applied to other population segments not represented in the included studies. To enhance the generalizability of the findings, future research should incorporate more varied samples in terms of age, sex, gender, ethnicity, and overall health, and should employ a mix of subjective and objective sleep measures.

Hence, increasing the public awareness for the importance of sleep in health, through preventive campaigns, could facilitate the collection of large amounts of data and permit to act preventively against insufficient sleep, bad sleep habits and untreated sleep disorders, which can represent risk factors for cognitive impairment, dementia and AD. Pharmacological and non-pharmacological sleep-based interventions can also demonstrate the potential of enhancing sleep quality to potentially counteract AD neurodegenerative processes in older adults. Hypnotics, mindfulness, cognitive behavioral therapy, and stress management approaches exhibited efficacy in improving sleep quality and health outcomes in adults [37]. Light therapy, particularly blue light exposure in the early evening, has been observed to enhance sleep efficiency in older adults, including those with AD [38]. Notably, both sleep duration and quality play significant roles in cognitive function. This emphasizes the importance of optimizing both sleep duration and quality in interventions targeting cognitive decline. In conclusion, sleep-based interventions present a promising approach for improving sleep quality and potentially decelerating AD progression in older adults. These findings underscore the potential of sleep-focused interventions in mitigating cognitive decline and enhancing overall health outcomes in the aging population.

## Data Availability

No new data were created or analysed in this study. Data sharing is not applicable to this article.

## Abbreviations

AD: Alzheimer’s disease
Aβ: β-amyloid
NREM: Non-rapid eye movement
ICD-9: International Classification of Diseases − 9th Edition
MCI: Mild cognitive impairment
CIND: Cognitive impairment and no dementia
FDA: Food and Drug Administration
SWS: Slow-wave sleep
REM: Rapid Eye Movement
OSA: Obstructive Sleep Apnea
PSQI: Pittsburgh Sleep Quality Index
JSEQ: Jenkins Sleep Evaluation Questionnaire
JSS: Jenkins Sleep Problems Scale
DWRT: Delayed Word Recall Test
MMSE: Mini Mental State Examination
HRS: Health and Retirement Study
DMS: Difficulty maintaining sleep
DIS: Difficulty initiating sleep
EMA: Early morning awakening
EDS: Excessive daytime sleepiness
OR: Odds ratio
CI: Confidence interval
NPI-Q: Neuropsychiatric Inventory Questionnaire
BVRT: Benton Visual Retention Test
CAMDEX: Cambridge Mental Disorders of the Elderly Examination
CDR: Clinical Dementia Rating
CERAD: Consortium to Establish a Registry for Alzheimer’s Disease
CERAD-e: Consortium to Establish a Registry in Alzheimer’s Disease battery
AD8: Dementia Screening Interview
DST: Digit Symbol Test
FAB: Frontal Assessment Battery
IADL: Instrumental Activities of Daily Living
IST: Isaacs Set Test
CERAD-K: Korean version of the Consortium to Establish a Registry for Alzheimer’s Disease Assessment Packet
MINI: Mini-International Neuropsychiatric Interview
3MS: Modified Mini-Mental State
TICS-m: Modified Telephone Interview for Cognitive Status
NCGG-FAT: National Center for Geriatrics and Gerontology Functional Assessment Tool
NYU PRT: New York University Paragraph Delayed Recall
SM-MMSE: Singapore-Modified Mini-Mental State Examination
MIS: The Memory Impairment Screen
NSD: Non-apnea sleep disorder
SWA: Slow-wave activity
PFC: Prefrontal cortex
DSM: Diagnostic and Statistical Manual of Mental Disorders
PRISMA: Preferred Reporting Items for Systematic Reviews and Meta-Analyses
SD: Standard Deviation
aHR: Adjusted Hazard Ratios, SDB: sleep-disordered breathing
ApoE: apolipoprotein E
